# Hourly test reference weather data in the changing climate of Finland for building energy simulations

**DOI:** 10.1016/j.dib.2015.04.026

**Published:** 2015-05-19

**Authors:** Kirsti Jylhä, Kimmo Ruosteenoja, Juha Jokisalo, Karoliina Pilli-Sihvola, Targo Kalamees, Hanna Mäkelä, Reijo Hyvönen, Achim Drebs

**Affiliations:** a Finnish Meteorological Institute, P.O. Box 503, 00101 Helsinki, Finland; b Aalto University School of Engineering, P.O. Box 14100, 00076 Aalto, Finland; c Tallinn University of Technology, Ehitajate tee 5, 19086 Tallinn, Estonia

## Abstract

Dynamic building energy simulations need hourly weather data as input. The same high temporal resolution is required for assessments of future heating and cooling energy demand. The data presented in this article concern current typical values and estimated future changes in outdoor air temperature, wind speed, relative humidity and global, diffuse and normal solar radiation components. Simulated annual and seasonal delivered energy consumptions for heating of spaces, heating of ventilation supply air and cooling of spaces in the current and future climatic conditions are also presented for an example house, with district heating and a mechanical space cooling system. We provide details on how the synthetic future weather files were created and utilised as input data for dynamic building energy simulations by the IDA Indoor Climate and Energy program and also for calculations of heating and cooling degree-day sums. The information supplied here is related to the research article titled “Energy demand for the heating and cooling of residential houses in Finland in a changing climate” [1].

**Specifications Table**

**Table:** 

Subject area	Civil engineering; Atmospheric physics
More specific subject area	Meteorology; Climatology; Building energy simulations
Type of data	Tables, figure, equations
How data was acquired	Meteorological data archives of the Finnish Meteorological Institute; the WCRP CMIP3 Multi-Model data archive; the IDA Indoor Climate and Energy simulation program.
Data format	Analysed and processed output data
Experimental factors	Quality control for the observational weather data and for the climate model data was conducted before the actual research work. The months for TRY2012 were selected using the Finkelstein-Schafer parameters that were weighted according to the importance of the individual climate variables for the building energy consumption in Finland.
Experimental features	Estimates for changes in the climate variables were based on CMIP3 multi-model data; and estimates for changes in delivered energy consumption were obtained using the IDA Indoor Climate and Energy simulation program.
Data source location	Data is given within this article.
Data accessibility	Data is given within this article.

**Value of the data**

• Future demand of heating and cooling energy is determined by future climate change.
• Future delivered energy consumption is also affected by the technical solutions employed for heating and cooling.
• It is essential that test reference year weather data used in building energy simulations is representative for the prevailing climatic conditions.
• Studying a wide ensemble of climate models is necessary in order to have a reasonable picture of the anticipated climate change.
• A procedure is presented for the development of synthetic future hourly-mean solar radiation data with the aid of climate model projections for global solar radiation and observed average partition between direct and diffuse radiation components as a function of global radiation.

## 1 Data, materials and methods

### 1.1 Current values and future changes in test reference meteorological data

The annual and seasonal mean values of air temperature (*T*), wind speed (*W*) and relative humidity (RH) according to the test reference year data set (TRY2012) for the current climate in southern Finland (the Helsinki–Vantaa weather station) are shown in Table 1. Table 2 gives the corresponding data for global (*G*) and diffuse (*F*) solar radiation on a horizontal surface as well as for direct solar radiation normal to the solar beam (*D*^norm^). As justified by [1], the year is divided into three periods: the major heating season (Nov–Mar), the cooling season (May−Aug), and the intermediate season (Apr, Sep−Oct). The 30-year averages and inter-annual standard deviations in 1980–2009 are likewise given in Tables 1 and 2.

Based on single-sample *t*-tests, the annual, seasonal (Table 1) and monthly means of *T* in TRY2012 do not significantly deviate from the corresponding 30-year averages. The same is true for the annual and all or most seasonal means of RH and *G*, for the annual mean of *D*^norm^ and for the major heating season means of *W* and *F* (Tables 1 and 2). In contrast, the 30-year average annual and most seasonal means of *W* and *F* and the monthly means of all the radiation components are less accurately captured by TRY2012. Even so, the differences between the TRY2012 means and the 30-year means of *G*, *F* and *D*^norm^ are smaller than one standard deviation of inter-annual variability for all months of the cooling and intermediate seasons, except of October for *G*. Comparisons of the cumulative frequency distributions of *T*, *F* and *D*^norm^ based on TRY2012 to those derived from the whole 30-year period (Fig. 3 in [1]) likewise indicate that the TRY2012 data set is climatologically representative, i.e., it can describe weather conditions typical in 1980−2009.

Tables 1 and 2 additionally show the estimated changes in the 30-year averages of the climatic variables for year 2100 under three alternative greenhouse gas scenarios (B1, A1B and A2). For details on how the estimates were made, see Sections 1.2 and 1.3. A future change given in bold indicates that, in terms of the observed inter-annual variations, the future test reference year TRY2100 value deviates significantly (with *p*<0.05 for single-sample *t*-tests) from the TRY2012 value. Significant increases are projected to take place by 2100 in the annual, major heating season, intermediate season and cooling season means of *T* and in the annual and major heating season means of *W* and RH. Significant reductions are estimated to occur in the major heating season means of *G*, *F* and *D*^*norm*^. The implications of the climatic changes in Tables 1 and 2 for the building energy demand are discussed by [1].

### 1.2 Development of future hourly temperature data

The future changes in annual, seasonal (Table 1) and monthly means of *T* were calculated as multi-model means between the baseline period 1980−2009 and 30-year periods centred at 2030, 2050 and 2100. For developing future hourly time series of temperature, to be used as input data for dynamic building energy simulations in [1], the multi-model mean climate change projections were combined with test reference weather data for the current climate. We employed the method denoted M2 in the paper by Räisänen and Räty [2]. Besides the projected changes in monthly mean *T*, this method takes into account changes in the standard deviation (*σ*_*T*_) of daily mean temperature. The number of climate models with available output data was 19 for monthly means and 10 for daily means (Table 3). We additionally assumed that, although temperature fluctuates more strongly on an hourly than on daily time scales, the percentage changes in variability can be considered practically similar on both time scales. This implies that for the future test reference year TRY2050, for example, temperatures at a 1-hour time step (*t*) can be approximated using the following equation:

(1) $$T_{\mathrm{TRY2050}}(t)=T_{\mathrm{TRY2012}}(t)+\Delta \bar{T}+\left(\frac{\sigma_{T_{2035-2064}}}{\sigma_{T_{1980-2009}}}-1\right)\left(T_{\mathrm{TRY2012}}(t)-{\bar{T}}_{\mathrm{TRY2012}}\right)$$

where *T*_TRY2012_ denotes the hourly temperature value in the current reference year, $\Delta \bar{T}$ is the projected change (°C) in the 30-year average monthly mean temperature from the current to the future time period, *σ*_T,1980–2009_ and *σ*_T,2035–2064_ are the standard deviations of daily temperatures in the climate model simulations during these two periods, and is the mean temperature of the calendar month considered, as given by the TRY2012 set.

Using the terminology of Belcher et al. [5], the morphing procedure (Eq. (1)) involves a combination of a shift and a stretch (or actually a shrinkage, since *σ*_*T*_ is projected to decrease rather than increase in Finland, particularly in winter). The method is unlikely to realistically reproduce the high frequency of temperatures close to 0 °C during the melting of snow, but this drawback is not critical in the current study.

### 1.3 Development of future hourly solar radiation data

The future changes in annual, seasonal (Table 2) and monthly means of global solar radiation on a horizontal surface (*G*) were estimated based on output from 18 climate models (Table 3). For developing synthetic future hourly files of *G*, we applied the method denoted by M1 in [2], except that the simulated relative rather than the absolute changes in *G* were considered, i.e., the time series of observed *G* were multiplied by the model-projected relative time-mean changes. For the direct (*D*) and diffuse (*F*) components of solar radiation separately, no information was provided by the climate models. In order to estimate how the projected changes in *G* would apportion between *D* and *F*, we took an empirical approach and utilised an observed average partition between the radiation components.

**Fig. 1:**
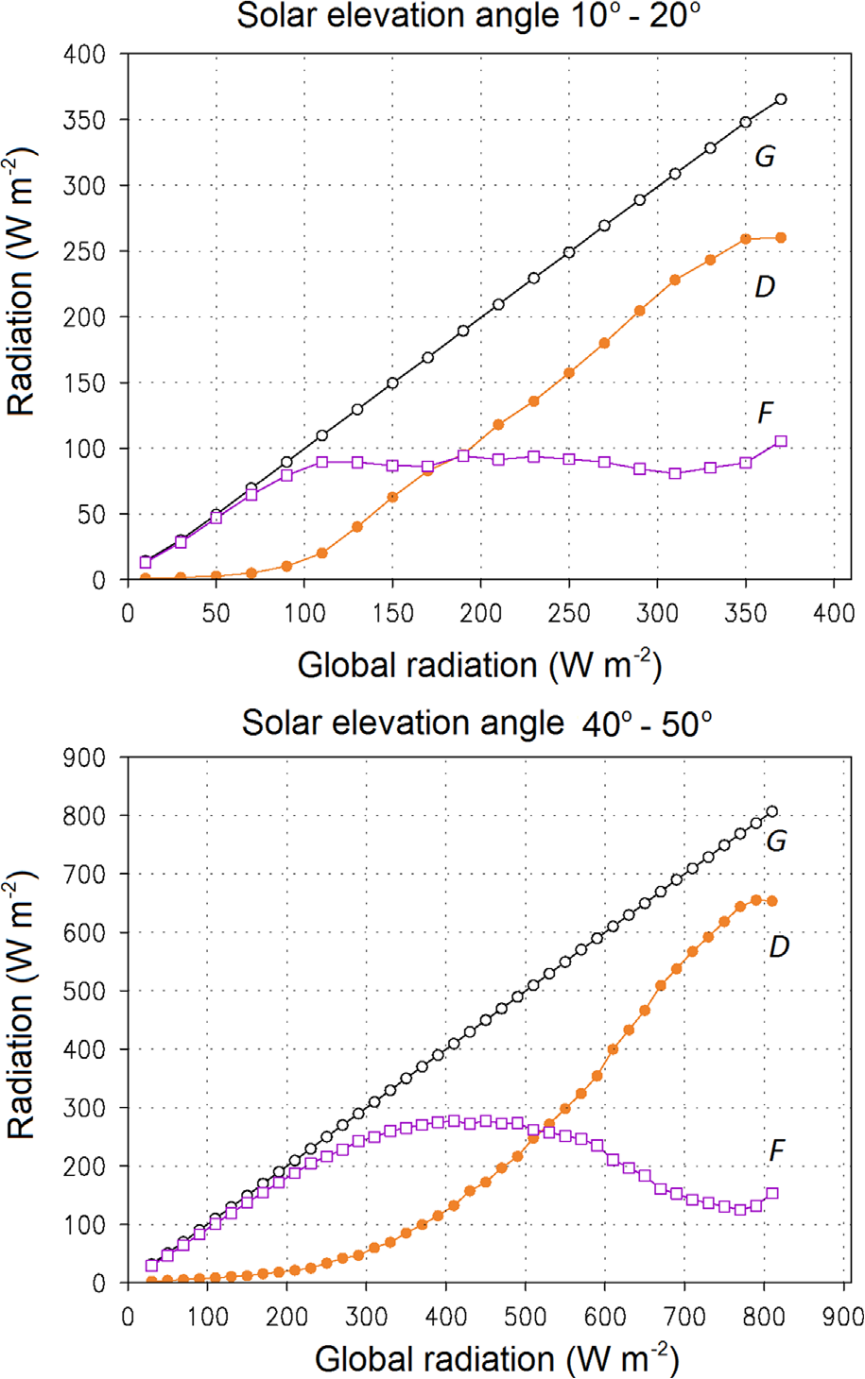
Observed average partition between hourly-mean direct (*D*, solid dots) and diffuse radiation (*F*, open squares) as a function of global solar radiation (*G*, open circles) under two categories of solar elevation angle: 10–20° (top) and 40–50° (bottom). The relationships are based on observations at three measurement stations in Finland (Helsinki–Vantaa, Jyväskylä and Sodankylä) in 1980–2009. Note differences in the vertical scales. All radiation fluxes are measured on a horizontal surface.

A collection of more than 260,000 radiation flux recordings from the period 1980–2009 at three weather stations in Finland was first classified based on the solar elevation angle (*α*). Data for *D* and *F* were then categorised depending on *G*. An approximate relation was thereby found between the *D*-to-*F* ratio and *G*. If *G* was high at a given α, the *D*-to-*F* ratio likewise appeared to be high. By contrast, if only a small amount of global radiation was measured in the same category of α, the ratio was low, suggesting a cloudy weather. As a rule of thumb, *F* was nearly equal to *G* up to a certain threshold that was dependent on *α* (Fig. 1). Beyond that threshold, the surplus of global radiation was received as direct radiation. Conversely, decreases in global radiation typically first materialized as reductions in direct radiation. Only at small values of *G* (and small *D*-to-*F* ratios) did the decreases in *G* occur as drops in *F*.

Climate models projected *G* to decline in Finland in the future in all seasons apart from summer and early autumn (Table 2, [6]). Assuming that the above-discussed empirical dependencies between *G*, *D* and *F* are also approximately valid in the future, the hourly solar radiation components in the TRY2050 data set were estimated as follows:

(2) $$G_{\mathrm{TRY2050}}(t)=G_{\mathrm{TRY2012}}(t)\cdot \left(1+\frac{\Delta \bar{G}}{100}\right)$$

(3a) $$D_{\mathrm{TRY2050}}\left(t\right)=0,\;\mathrm{if}\;D_{\mathrm{TRY2012}}\left(t\right)=0$$

(3b) $$D_{\mathrm{TRY2050}}(t)=max\left\{0,\;D_{\mathrm{TRY2012}}(t)+G_{\mathrm{TRY2050}}(t)-G_{\mathrm{TRY2012}}(t)\right\},\;\mathrm{if}\;D_{\mathrm{TRY2012}}\left(t\right)>0$$

(4) $$F_{\mathrm{TRY2050}}\left(t\right)=G_{\mathrm{TRY2050}}\left(t\right)-D_{\mathrm{TRY2050}}\left(t\right)$$

(5) $$D_{TRY2050}^{norm}(t)=D_{TRY2012}^{norm}(t)\cdot \frac{D_{\mathrm{TRY2050}}(t)}{D_{\mathrm{TRY2012}}(t)}$$

where $\Delta \bar{G}$ is the percentage change in the multi-model mean 30-year average of monthly global radiation from the current to the future time period, and *D*^norm^ is the direct solar radiation normal to the solar beam. Corresponding equations were used for TRY2030 and TRY2100.

As indicated by Eqs. (2)–(5), the projected changes in *G* were primarily applied to *D* and only secondarily to *F*. Only in overcast situations with no direct radiation was the modelled change in global radiation entirely allotted to diffuse radiation. At other times, *D* was chiefly modified by the same absolute amount of energy as *G*. However, if *G* was projected to decrease by an amount larger than the baseline value of *D*, the surplus reduction was taken from in *F*. The direct radiation normal to the solar beam was finally scaled by the same factor as *D*. Note that the number of hours with no solar radiation remained constant.

The empirical method ignores a feature evident in Fig. 1, namely that at very high values of *G*, the dependence of *F* and *D* on *G* is rather complicated. Otherwise, the method is in agreement with simple physical reasoning, although certainly only approximately. Besides us, the problem of missing climate model output data for the solar radiation components was also encountered by Belcher et al. [5]. In order to construct design weather data for future climates, they ended up using the same monthly mean scaling factor for *F* as for *G*. Here, by contrast, we made smaller monthly-average subtractions (in summer, smaller additions) to *F* than to *G*. Besides based on Fig. 1, this can be justified by climate model projections for the total cloud cover in Finland: it is expected to increase in winter and remain almost unchanged in summer [7]. Note that the IDA-ICE building energy simulation tool uses *F* and *D*^norm^ as input data rather than cloud cover or sunshine duration.

### 1.4 Projected changes in delivered energy consumption for district heating and space cooling electricity (case B)

The hourly test reference weather files based on data given in Tables 1 and 2 and described in more detail in Sections 1.2 and 1.3 were used as input data for the IDA Indoor Climate and Energy simulation tool [1]. Table 4 shows the annual and seasonal delivered energy consumptions for heating of spaces, heating of ventilation supply air and cooling of spaces in the example house considered by [1] for case B, with district heating and a mechanical space cooling system. For the years 2030, 2050 and 2100, the values are based on the A1B scenario. The corresponding monthly values using the TRY2012 and TRY2100 data are shown in Fig. 6 of [1].

### 1.5 Heating and cooling degree-day sums

Besides as input data for dynamic building energy simulations, the current and future test reference temperature files were employed by [1] to calculate heating and cooling degree-day sums. In that method, the impacts of the internal heat gains are taken into account by using an effective indoor temperature *T*_*e*_ that is lower than the actual target indoor temperature, the latter being 21.5 °C in most rooms of the example house (Fig. 1 in [1]). For heating degree days to accumulate, the daily mean outdoor temperature needs to fall below a threshold *T*_*c*_. Cooling degree days are in turn calculated at a temperature above the effective indoor temperature. Their monthly and yearly sums across days *j* are given as follows:

(6) $$HDD=\sum_{i}\max\left(0,T_{\mathrm{eh}}-{\bar{T}}_{d}(i)\right)\;\;\mathrm{if}\;{\bar{T}}_{d}(i)<T_{c}$$

(7) $$CDD=\sum_{i}\max\left(0,{\bar{T}}_{d}(i)-T_{\mathrm{ec}}\right)$$

where the effective indoor temperature for heating (*T*_*eh*_) is 17 °C [8] and for cooling (*T*_*ec*_) 18 °C [9]. In Eq. (6), the threshold *T*_*c*_ is higher (12 °C) at the beginning of the heating period in autumn (up to December) than at the end of the season in spring (10 °C) [8]. The annual rhythm of *T*_*c*_ approximates the impact of the more abundant solar radiation in spring than in autumn in Finland.

### 1.6 The net present value

In addition to the heating and cooling (Table 4), delivered energy is consumed in buildings for other purposes, such as domestic hot water and lighting (Table 1 in [1]). Based on the estimated changes in the yearly total delivered energy consumption, [1] assessed the net present value (NPV) of the direct effect of the changing climate on the energy cost per square metre. NPV can be calculated with the well-known equation [10]

(8) $$NPV(i,N)=\sum_{t=0}^{N}\frac{R_{t}}{{\left(1+i\right)}^{t}}$$

where *R*_*t*_ denotes the change in the annual energy cost (€) relative to the baseline (TRY2012), *i* is the fractional discount rate, *t* the time in years and *N* is the total number of years (*N*=88 for TRY2100).

## Figures and Tables

**Table 1:** Annual and seasonal mean values of temperature, wind speed and relative humidity based on observations, and model-based estimates for their changes. For each variable, first shown are the long-term (1980–2009) mean, the inter-annual standard deviation in 1980−2009 and the TRY2012 mean. The projected changes in the TRY2100 data sets, relative to the TRY2012 mean, are denoted separately for the B1, A1B and A2 scenarios. Statistically significant (with *p*<0.05) differences between the TRY2012 means and the 1980–2009 averages, as well as between the TRY2100 and TRY2012 means, are given in bold.

Variable	Annual	Major heating season	Intermediate season	Cooling season
Temperature (°C)				
1980–2009 mean	5.3	−3.0	6.8	14.5
1980–2009 stdev	1.0	1.9	1.1	0.9
TRY2012	5.6	−2.6	7.1	14.6
Changes by 2100 (°C)				
TRY2100_B1	**2.7**	**3.5**	**2.3**	**2.0**
TRY2100_A1B	**3.9**	**5.0**	**3.3**	**3.0**
TRY2100_A2	**5.1**	**6.6**	**4.5**	**3.8**
Wind speed (m s^−1^)				
1980–2009 mean	4.0	4.2	3.9	3.7
1980–2009 stdev	0.3	0.4	0.3	0.3
TRY2012	**4.1**	4.3	**4.1**	**4.1**
Changes by 2100 (%)				
TRY2100_B1	2	3	1	1
TRY2100_A1B	**3**	**4**	2	1
TRY2100_A2	**4**	**6**	2	2
Relative humidity (%)				
1980–2009 mean	79	86	79	70
1980–2009 stdev	2	2	3	3
TRY2012	79	86	79	70
Changes by 2100 (% units)				
TRY2100_B1	**1**	**2**	0	1
TRY2100_A1B	1	**3**	0	−**1**
TRY2100_A2	**2**	**4**	1	−1

**Table 2:** Same as Table 1 but for global solar radiation on a horizontal surface, direct solar radiation normal to the solar beam and diffuse solar radiation on a horizontal surface.

Variable	Annual	Major heating season	Intermediate season	Cooling season
Global solar radiation (W m^−2^)				
1980–2009 mean	293	85	266	574
1980–2009 stdev	13	10	26	31
TRY2012	293	**76**	274	577
Changes by 2100 (%)				
TRY2100_B1	0	**−8**	1	1
TRY2100_A1B	**−**1	**−12**	0	1
TRY2100_A2	**−3**	**−16**	**−2**	**−**1
Normal direct solar radiation (W m^−2^)				
1980–2009 mean	327	117	303	609
1980–2009 CV (%)	11	24	21	11
TRY2012	340	**90**	**339**	**652**
Changes by 2100 (%)				
TRY2100_B1	0	**−18**	2	2
TRY2100_A1B	**−**1	**−25**	1	2
TRY2100_A2	**−5**	**−32**	**−**2	**−**1
Diffuse solar radiation (W m^−2^)				
1980–2009 mean	135	50	132	243
1980–2009 CV (%)	5	7	3	7
TRY2012	**129**	49	**128**	**230**
Changes by 2100 (%)				
TRY2100_B1	**−**1	**−4**	0	0
TRY2100_A1B	**−**1	**−6**	**−**1	0
TRY2100_A2	**−**2	**−9**	**−**1	0

**Table 3:** Global climate model output utilised in this study. 1st column, model acronym; columns 2--6, availability of data for monthly mean temperature (*T*), daily mean temperature (*T*_*d*_), global solar radiation (*G*), relative humidity (*RH*) and wind speed (*W*). For more information on the models, see the footnotes and Table 8.1 of [3].

Model acronym	*T*	*T*_*d*_	*G*	*RH*	*W*^a^
BCCR-BCM2.0	x	x	x	x	x
CGCM3.1(T47)	x		x	x	
CGCM3.1(T63)^b^	x	x	x	x	x
CNRM-CM3	x	x	x	x	x
CSIRO-MK3.0	x	x			x
ECHAM5/MPI-OM	x	x	x	x	x
ECHO-G	x		x		
GFDL-CM2.0	x		x		
GFDL-CM2.1	x	x	x		x
GISS-ER	x		x		
INM-CM3.0	x		x	x	
IPSL-CM4	x	x	x		x
MIROC3.2(HIRES)^b^	x	x	x		x
MIROC3.2(MEDRES)	x		x		
MRI-CGCM2.3.2	x	x	x		x
NCAR-CCSM3	x	x	x		
NCAR-PCM	x		x		
UKMO-HadCM3	x		x	x	
UKMO-HadGEM1	x		x		
Number of models	19	10	18	7	9

a Data for zonal and meridional daily wind components were available for the periods 1971−2000, 2046−2065 and 2081−2100 only. In order to assess changes in climatological means of the periods 2015−2044 (midpoint 2030), 2035−2064 (midpoint 2050) and 2085−2114 (midpoint 2100), relative to 1980−2009, linear interpolation and extrapolation were used.

b Model experiments under the SRES A2 are not available. In order to make the multi-model mean responses in *T*, *G*, RH and *W* comparable for all three GHG scenarios, surrogate data for the missing A2 runs were created by employing the pattern-scaling technique detailed in [4].

**Table 4:** Annual and seasonal delivered energy consumption (kWh m^−2^) for heating and cooling in the example house in the current climate conditions (TRY2012) and in the future, assuming the A1B scenario. District heating and a mechanical space cooling system (case B) are assumed.

	Delivered energy consumption (kWh m^−2^)			
	Annual	Heating season (Nov−Mar)	Intermediate season (Apr, Sep, Oct)	Cooling season (May−Aug)
TRY2012				
District heating of spaces	78.0	65.1	12.2	0.8
District heating of ventilation	11.5	9.6	1.7	0.2
Space cooling electricity	3.9	0.0	0.2	3.7

TRY2030_A1B				
District heating of spaces	71.0	60.0	10.5	0.5
District heating of ventilation	9.7	8.3	1.3	0.1
Space cooling electricity	4.8	0.0	0.3	4.5

TRY2050_A1B				
District heating of spaces	65.1	55.6	9.2	0.3
District heating of ventilation	8.4	7.2	1.1	0.1
Space cooling electricity	5.3	0.0	0.3	5.0

TRY2100_A1B				
District heating of spaces	55.7	48.3	7.2	0.1
District heating of ventilation	6.4	5.6	0.7	0.0
Space cooling electricity	6.5	0.0	0.5	6.0

## References

[1] Jylhä K., Jokisalo J., Ruosteenoja K., Pilli-Sihvola K., Kalamees T., Seitola T., Mäkelä H., Hyvönen R., Laapas M., Drebs A. (2015). Energy demand for the heating and cooling of residential houses in Finland in a changing climate. Energy Build.

[2] Räisänen J., Räty O. (2013). Projections of daily mean temperature variability in the future: cross-validation tests with ENSEMBLES regional climate simulations. Clim Dyn.

[3] IPCC, Change S., Solomon D., Qin M., Manning Z., Chen M., Marquis K.B., Averyt M., Tignor, Miller H.L. (2007). Climate Change 2007: the physical science basis. Contribution of Working Group I to the Fourth Assessment Report of the Intergovernmental Panel on Climate.

[4] Ruosteenoja K., Tuomenvirta H., Jylhä K. (2007). GCM-based regional temperature and precipitation change estimates for Europe under four SRES scenarios applying a super-ensemble pattern-scaling method. Clim. Change.

[5] Belcher S.E., Hacker J.N., Powell D.S. (2005). Constructing design weather data for future climates. Build. Serv. Eng. Res. Technol..

[6] Ruosteenoja K., Räisänen P. (2013). Seasonal changes in solar radiation and relative humidity in Europe in response to global warming. J. Clim..

[7] Jylhä K., Ruosteenoja K., Räisänen J., Venäläinen A., Tuomenvirta H., Ruokolainen L., Saku S., Seitola T. (2009). Arvioita Suomen muuttuvasta ilmastosta sopeutumis-tutkimuksia varten. ACCLIM-hankkeen raportti 2009 (The changing climate in Finland: estimates for adaptation studies. ACCLIM project report 2009).

[8] Vajda A., Venäläinen A., Tuomenvirta H., Jylhä K. (2004). An estimate about the influence of climate change on heating energy demand in Hungary, Romania and Finland. Idojárás.

[9] Jäger J. (1983). Climate and Energy Systems: A Review of Their Interactions.

[10] Boardman A., Greenberg D., Vining A., Weimer D. (2010). Cost-Benefit Analysis.

